# A Novel PCR Method for Detecting ACE Gene Insertion/Deletion Polymorphisms and its Clinical Application

**DOI:** 10.1186/s12575-020-00140-6

**Published:** 2021-01-07

**Authors:** Xue-min Yang, Jian-ping Liang, Xiao-juan Huang, Xiang-rong Wang, Yang Sun, Chen Dong, Ya-li Cui, Wen-li Hui

**Affiliations:** 1 The College of Life Science, Northwest University, Xi’an, 710069 Shanxi China; 2 Shanxi Provincial Engineering Research Center for Nano-Biomedical Detection, Xi’an, 710077 Shanxi China; 3 Data Center of Shaanxi Provincial People’s Hospital, Xi’an, 710069 Shanxi China

**Keywords:** Insertion/deletion polymorphisms, Direct-polymerase chain reaction (direct-PCR), Rapid detection, Gold magnetic nanoparticle lateral flow assay

## Abstract

**Background:**

Angiotensin-converting enzyme (ACE) plays a major role in blood pressure regulation and cardiovascular homeostasis. The wide distribution and multifunctional properties of ACE suggest it’s involvement in various pathophysiological conditions.

**Results:**

In this study, a novel visual detection method for ACE I/D polymorphisms was designed by integrating direct PCR without the need for DNA extraction using gold magnetic nanoparticles (GMNPs)-based lateral flow assay (LFA) biosensor. The entire detection procedure could enable the genotyping of clinical samples in about 80 min. The detection limit was 0.75 ng and results could be obtained in 5 min using the LFA device. Three hundred peripheral blood samples were analyzed using the direct PCR-LFA system and then verified by sequencing to determine accuracy and repeatability. A clinical preliminary study was then performed to analyze a total of 633 clinical samples.

**Conclusions:**

After grouping based on age, we found a significant difference between the genotypes and the age of patients in the CHD group. The introduction of this method into clinical practice may be helpful for the diagnosis of diseases caused by large fragment gene insertions/deletions.

## Background

At present, most of the detection methods for large fragment insertion/deletion (I/D) polymorphism are based on traditional polymerase chain reaction and electrophoresis. Rigat et al. were the first to describe a method for the detection of I/D polymorphisms using polymerase chain reaction (PCR) and agarose gel electrophoresis in 1992 [1, 2]. Other techniques including real-time polymerase chain reaction (RT-PCR) [3–5], high resolution melting (HRM) [5, 6], polymerase chain reaction-restriction fragment length polymorphism (PCR-RFLP) [7] and microchip electrophoresis (ME) system [8] have also been used to detect I/D polymorphism. However, these techniques require tedious experimental procedures and expensive and sophisticated instruments that may not be available in clinical institutions. In addition, most of these detection systems are based on fluorescence for detection and analysis or have high requirements for sample purity. These requirements make it inconvenient and difficult for the rapid detection of I/D polymorphisms from whole blood. Hence, an easy-to-operate and affordable onsite technique for genotyping with high efficiency is required.

We used ACE I/D polymorphisms as a genotyping model in this study. We designed a sensitive, rapid, and cost-effective method for I/D polymorphism detection. Angiotensin-converting-enzyme plays an essential role in two physiological systems, one leading to the production of angiotensin II and the other for the degradation of bradykinin. ACE metabolizes bradykinin, which is a strong vasodilator, forming the inactive metabolite bradykinin 1–5. ACE can also metabolizes neurokinins, which plays a key role in the transmission of pain, regulation of emotions, and alteration of inflammatory and immune responses. The broad distribution and multifunctionality of these peptides suggest that ACE may be involved in the development of several diseases [9]. The ACE gene is located in intron 16 on chromosome 17 in humans. A 287 bp insertion/deletion (I/D) polymorphism [2, 10], results in three genotypes: II (insertion homozygote), ID (insertion-deletion heterozygote) and DD (deletion homozygote) [11]. Several studies have demonstrated that this polymorphism is associated with cardiovascular and cerebrovascular diseases [12, 13], while other studies have failed to find an association [14–18]. However, ACE gene polymorphisms has been demostrated to guide the development of therapeutic drugs [19, 20]. Hence, the detection of ACE gene polymorphisms can guide the rational use of drugs in hypertensive patients with different ACE genotypes.

In our previous study, we established a lateral flow assay (LFA) assembled with GMNPs that relies on immune hybridization reactions for detecting SNPs, such as MTHFR C677T, which enables the typing of genomic DNA [21, 22]. In this study, we demonstrate a Direct PCR LFA system, which amplifies nucleic acids without the need for DNA extraction and can be used for detecting I/D polymorphisms of large fragments. Samples from fresh whole blood were first treated with NaOH, which facilitates DNA release from whole blood for direct amplification and eliminates PCR inhibits, such as hemoglobin and enzymes that degrade DNA [23]. Accuracy was determined in clinical samples by comparing the genotyping results generated from the Direct PCR LFA method with DNA sequencing. The Direct PCR LFA method showed excellent specificity, sensitivity, and robustness for detecting ACE gene I/D polymorphism.

## Results and Discussion

### Principles of the Direct PCR- LFA System

**Fig. 1:**
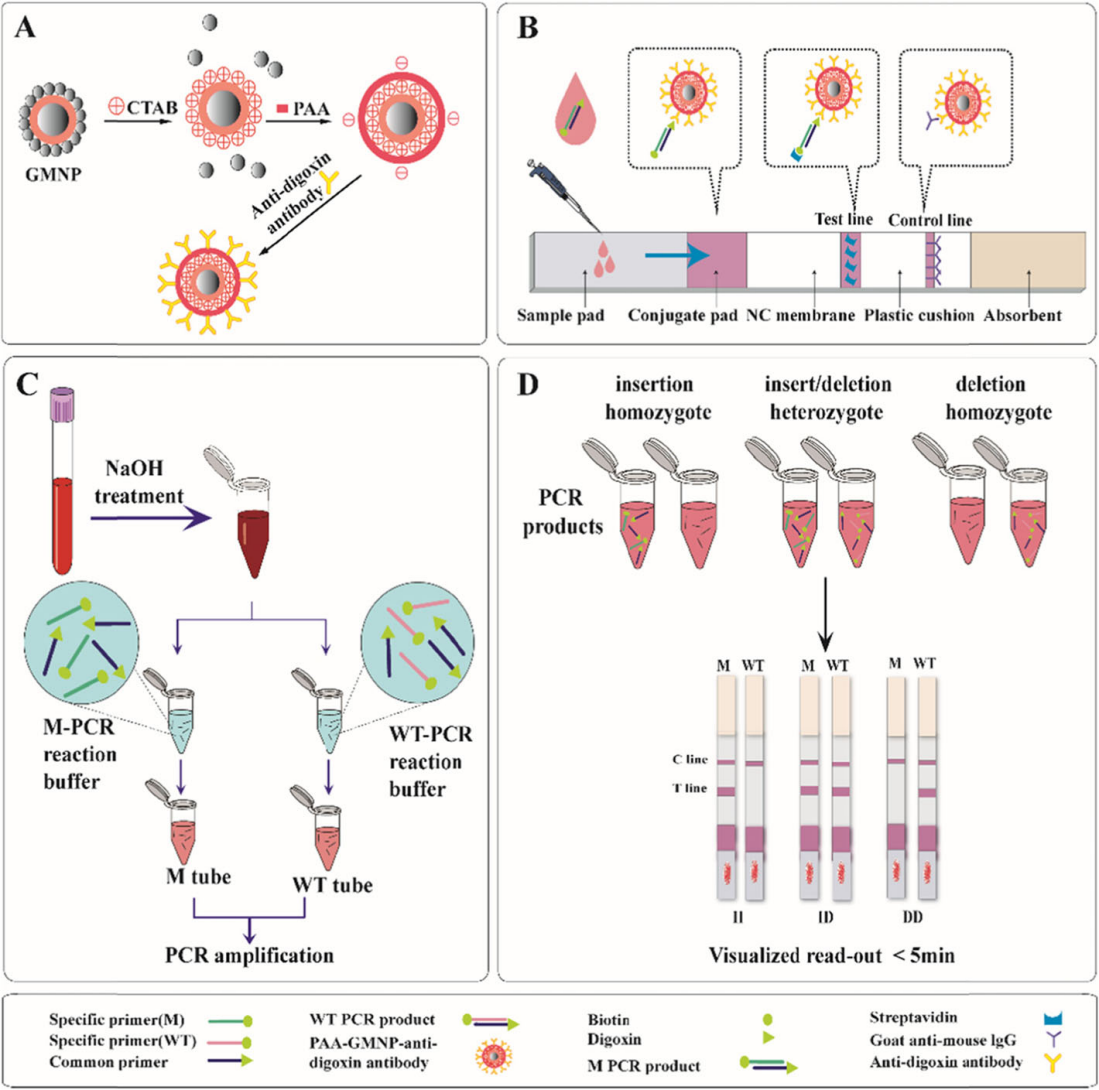
Schematic diagram of the Direct PCR-LFA system. **a** Schematic illustration for the GMNP surface modification process. **b** Structure of the labeled lateral flow device. **c** Sample preparation and target amplification. **d** Results based on visual inspection of bands in the C/T lines

To detect ACE I/D polymorphisms, we established the Direct PCR-LFA system. The principles of the Direct PCR-LFA system is schematically illustrated in Fig. 1.

GMNPs were synthesized based on the methods described previously [24, 25]. The GMNPs were functionalized with cetyltrimethylammonium bromide (CTAB) surfactant, followed by modification of the polyacrylic acid (PAA) and then conjugation of anti-digoxin antibody through 1-[3-(dimethylamino)propyl]-3-ethylcarbodiimide hydrochloride chemistry based on a previous method [26, 27] (Fig. 1a). The LFA device consisted of a strip composed of five overlapping pads, i.e., sample pad, conjugate pad, nitrocellulose membrane, absorbent pad, and plastic cushion (Fig. 1b). In brief, goat anti-mouse IgG and streptavidin were pre-immobilized in a control line (C line) and a test line (T line) respectively on a porous nitrocellulose membrane using the BioJet HM3010 dispenser (BioDot Inc., California, U.S.A.). Then, the probe solution containing the poly-acrylic acid (PAA) modified gold magnetic nanoparticles (PGMNPs) conjugated with anti-digoxin antibody was dispensed onto the conjugate pad of the LFA strips. The strips were dried and stored in a sealed aluminum foil bag at room temperature until required.

Because heparin has an inhibitory effect on PCR [28], we tested and verified the effect of heparin and EDTA on amplification, and obtained the same results. Therefore, the whole blood sample is required to be collected in a preservation tube containing EDTA. Fresh whole blood samples were treated with NaOH solution, followed by PCR amplification using two tubes with the same prepared blood template (Fig. 1c). After PCR amplification, the PCR products in the two tubes were added onto the sample pad of the LFA strip and the results read visually within 5 min (Fig. 1d). For insertion homozygous samples (II), a distinct red band was observed on the T line of the strip from the PCR products of the M tube but not from the WT tube. For deletion homozygous samples (DD), only a red band was observed on the strip from PCR products of the WT tube but not the M tube. If red bands with similar intensities are observed on the T lines of both strips, this indicates an insertion/ deletion heterozygous sample (I/D).

In the whole experiment above, the successful amplification of PCR and the normal operation of LFA system are important steps to obtain correct experimental results. This method is designed to use double tubes and double channels to complete the deletion and insertion gene amplification at the same time. The T line has a dual role. It can read the test results and also serve as the internal quality control of the PCR system. In addition, the C line can judge the effectiveness of the LFA system.

Our Direct PCR-GoldMag LFA was faster compared to existing methods. The pretreatment of the samples with NaOH is crucial for this method and makes the whole testing procedure convenient and rapid. This is because, compared to other detection methods, the DNA purification step is eliminated in our assay with the help of NaOH treatment. This shortens the processing time from 1 to 2 h to a mere 5 min. Second, by using NaOH-treated blood samples for PCR, the problem of cross-contamination, which exists in traditional blood DNA purification processes is eliminated. Furthermore, various PCR inhibitors present in the samples such as hemoglobin, IgG, lactoferrin, and proteases are inactivated by NaOH treatment [29, 30]. When the direct PCR product is loaded onto the sample pad of the LFA strip, certain impurities such as hemoglobin in the blood are filtered by the sample pad and conjugate pad, hence the chromatography is not affected.

By introducing mismatches at the penultimate or antepenultimate at the 3′ end of the primers, primers specificity was enhanced. We designed several primers to determine the optimal primers to use. After ARMS-PCR, the LFA device was used to detect the PCR products. With the help of gold magnetic nanoparticles and LFA, accurate and rapid genotyping results could be generated by visual inspection of colors on the T and C lines. It takes only 5 min to obtain the results without the need for expensive or high-end instruments. We also demonstrated that our method was more convenient and superior to the classical agarose gel electrophoresis method. Hence, this method could be used in medical and hospital laboratories with limited resources for purchasing specialized equipment.

### Performance of the Direct PCR- LFA System

To determine the optimal assay conditions, optimization of whole blood direct amplification was performed under different PCR cycling conditions, i.e., annealing temperature, concentration of the primers, etc. were optimized using whole blood samples with the three different ACE genotypes. The magnetic signal at the T line showed the best amplification efficiency and specificity when the cycle number was 31 (Figure S1A) and the annealing temperature was 60 °C (Figure S1B). In addition, the best primer concentration was found to be 2.5 μM (Figure S1C).

**Fig. 2:**
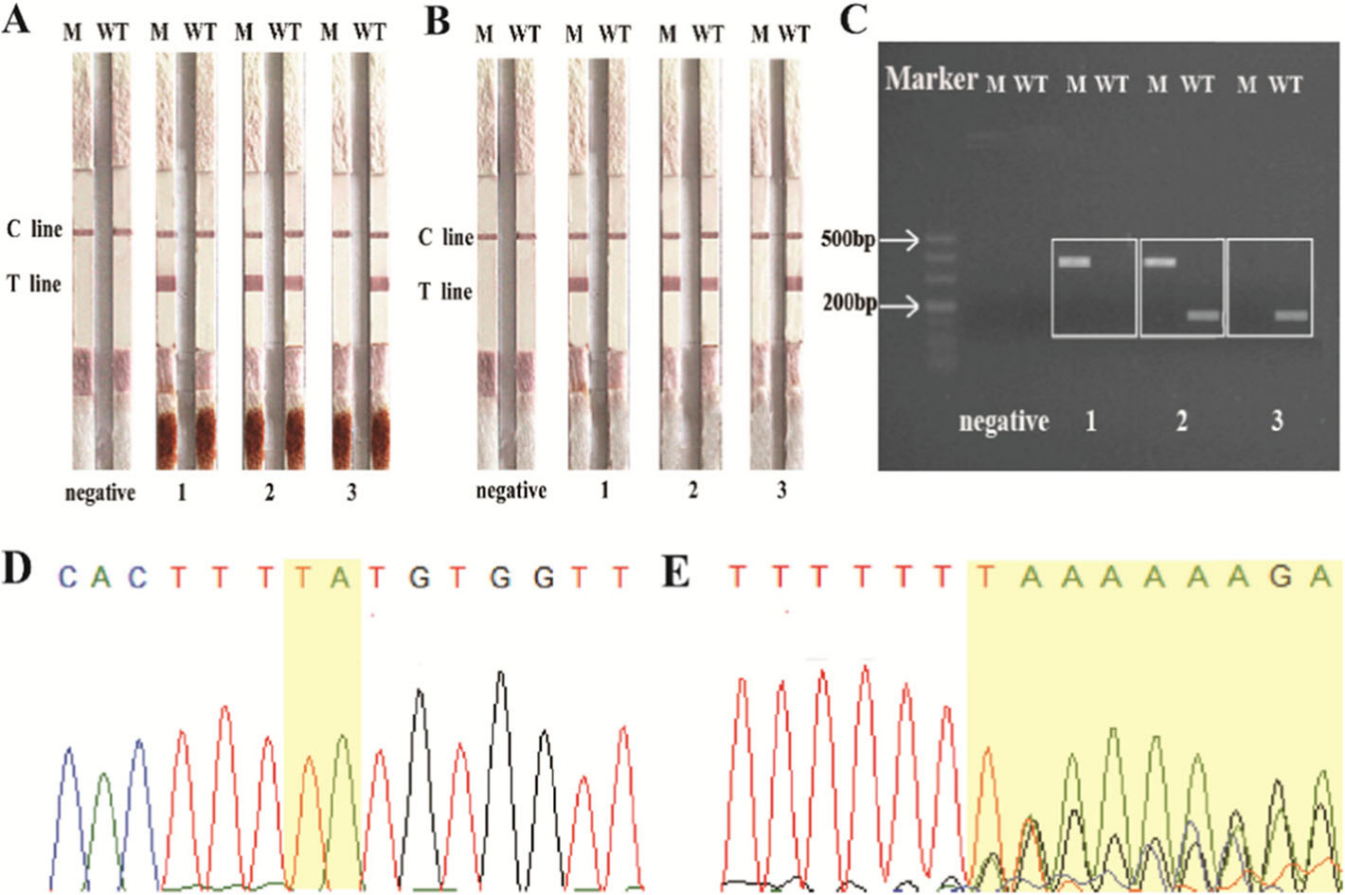
The specificity of the Direct PCR-LFA system. **a** Genotyping results of the whole blood Direct PCR-LFA system. M = M tube. WT = WT tube. 1: Homozygous insertion-deletion 2: Heterozygous 3: Homozygous deletion. **b** The genotyping result of PCR-LFA using DNA as the template. **c** The genotyping result of agarose gel electrophoresis. **d** DNA sequencing result of a Homozygous deletion sample. **e** DNA sequencing result of a Homozygous insertion sample

The specificity of the ACE genotyping was an important consideration. For specificity analysis, three known ACE genotypes (II, I/D, DD) were analyzed using the Direct PCR- LFA system (Fig. 2a). We also performed a crossover experiment to validate the primers. In addition, ACE genotyping results detected using the lateral flow assay (Fig. 2b) was compared with the classical agarose gel electrophoresis method (Fig. 2c). ACE gene I/D polymorphism is caused by either an insertion or deletion of the 287 bp Alu repeat. A gene fragment containing a repeat sequence cannot be accurately verified using the Sanger method. A homozygous deletion could be accurately measured after several rounds of sequencing, however, homozygous insertions are more difficult to accurately sequence (Fig. 2d-e).

To enhance primer specificity, we designed multiple primers to determine the optimal primers pairs to use in our assay (Table 1). In addition, we introduced mismatches in the penultimate or antepenultimate of the 3′ end of the primer for PCR. We observed that these modifications had an impact on the specificity of our method, as some allele-specific primers completely lost specificity upon removal of the mismatches [31]. This is important when analyzing clinical samples that require high specificity and accuracy. We then performed PCR and agarose gel electrophoresis using these primers and selected the most optimal primes to use in our assay. The deletion-specific primer sequence was 5′-AACCACATAAAAGTGACTGTATCGG-3′, and had a mismatch at the third of the 3′ end, while the insertion-specific primer sequence was 5′-TCGAGACCATCCCGGCTAAAAC-3′. Other primers that were synthesized were excluded due to poor specificity. The list of common primers is shown in Table 5. The common forward primer sequence was 5′-AAGGAGAGGAGAGAGACTCAAGCAC-3′.

**Table 1:** Multiple Primer Sequences used for assay optimization

Primer type	Primer name	Sequence (5′-3′)
Deletion-specific primer	F1	TTCTCTAGACCTGCTGCCTATACAG
	R1	CCATAACAGGTCTTCATATTTCCGG
	F2	TCTCTAGACCTGCTGCCTATACCGT
	R2	CCATGCCCATAACAGGTCTTCATAT
	F3	AAGGAGAGGAGAGAGACTCAAGCAC
	R3	GCGAAACCACATAAAAGTGACTGTATAG
	F4	AAGGAGAGGAGAGAGACTCAAGCAC
	R4	AACCACATAAAAGTGACTGTATCGG
Insertion-specific primer	F1	CTGGAGAGCCACTCCCATCCTTTCT
	R1	TCGAGACCATCCCGGCTAAAAC
	F2	GCCACTCCCATCCTTTCT
	R2	CCTGTAATCCCAGCACTTTG

### Performance of the PCR-GoldMag LFA

**Fig. 3:**
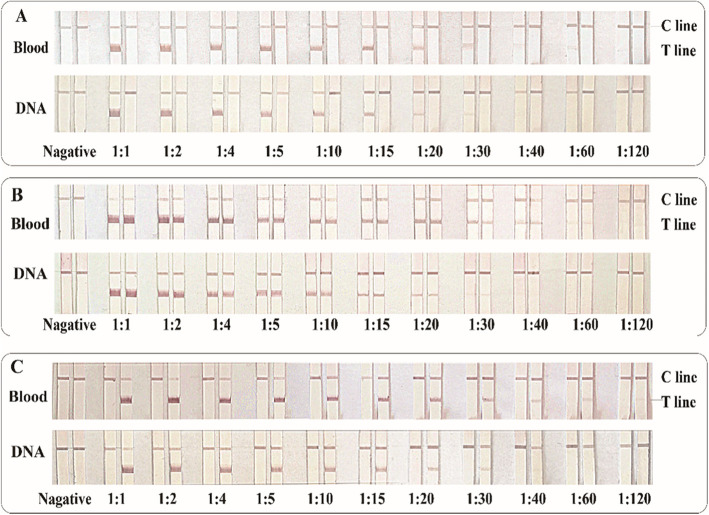
Comparison of results generated using gradient dilution of whole blood samples and nucleic acid samples (1:1–1:120). **a** Homozygous insertion sample (**b**) Insert/deletion heterozygous sample (**c**) Homozygous deletion sample

To evaluate the performance of the PCR-GoldMag LFA system, the sensitivity of LFA was evaluated using various amounts of whole blood samples with known ACE genotypes. Gradient dilutions of whole blood with physiological saline at 1:1, 1:2, 1:4, 1:5, 1:10, 1:15, 1:20, 1:30, 1:40, 1:60, 1:120 ratio were evaluated. Whole blood samples are compared with purified nucleic acid samples (Fig. 3). The LFA typing results were analyzed to determine the sensitivity of the whole blood direct PCR method. Simultaneously, we performed blood routine tests obtained from the People’s Hospital of Shaanxi Province (Table S1). The amount of nucleic acids in a white blood cell is approximately 5.6 × 10^− 9^ μg [32]. The results demonstrated that the minimum detection sensitivity was 0.75 ng.

Using our optimized conditions, PCR-GoldMag LFAs showed high specificity with no false-positive results and had a higher sensitivity. The detection limit of PCR-LFA could reach 0.75 ng of DNA, which was comparable to a PCR-DNA microassay commercial kit and had obvious advantages. As shown in Fig. 3, whole blood samples were more sensitive compared to purified DNA. At a dilution ratio of 1:60, bands could still be observed using whole blood, while from purified DNA, bands were fainter at the same dilution factor. For homozygous deletion samples, no bands appeared at a ratio of 1:15. This may be due to the loss of white blood cells during DNA extraction, while whole blood eliminated the DNA extraction step, and hence had no loss of white blood cells. The sensitivity was higher using whole blood samples compared to purified DNA. The capacity of antibody conjugation to nanoparticles is critical for the success of our assay. In general, the conjugating capacity of nanoparticles to antibodies at best is only about 50μgmg^− 1^. Because of the novel GoldMag nanoparticle structure (nanoflowers) [24], our antibody conjugation reached greater than 100μgmg^− 1^. This ensured the sensitivity and stability of our established PCR-lateral flow assay [24, 27].

### Clinical Applications

**Fig. 4:**
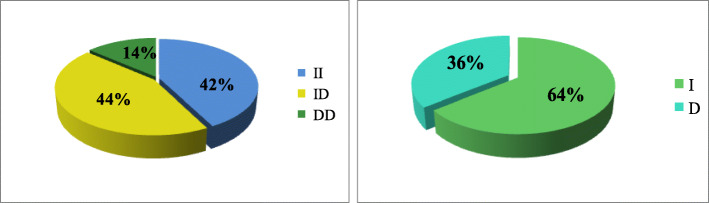
Pie chart for the genotype and allele frequency of ACE I/D polymorphism. **a** Genotype frequencies of ACE. **b** Allele frequencies of I and D alleles

To evaluate the reliability of our optimized PCR-GoldMag LFA method, the accuracy was further validated using an additional 300 clinical samples obtained from the Shaanxi Provincial People’s Hospital (Xi’an, China), with informed consent waived. Each sample was tested using our whole blood Direct PCR-GoldMag LFAs and agarose gel electrophoresis was used to compare our results. As shown in Table 2, the genotyping results were 100% consistent with the results obtained from agarose gel electrophoresis. In addition, we performed a Hardy-Weinberg equilibrium test on selected samples (Table 2) and analyzed the genotype and allele frequencies of the 300 cases (Fig. 4). The results were consistent with Hardy Weinberg’s law of equilibrium. It demonstrated that the selected groups had good group representation. The genotype frequencies of ACE II, ID, DD types were 42% (127 cases), 44% (132 cases), and 14% (41 cases) (Fig. 4a). The allele frequency for the I allele was 64% and the D allele was 36% (Fig. 4b). Representative genotyping results of the two methods are showed in Figure S2. In addition, we evaluated blood samples from cases with high bilirubin levels and low and high white blood cell counts (Figure S3). Our results demonstrated that the established method had good accuracy and reliability. We believe our method has wide application value in clinical practice.

**Table 2:** ACE genotyping results using whole blood samples between Direct PCR-LFA system and Agarose gel electrophoresis

	Genotype	Agarose gel electrophoresis			Total	Agreement
		ACE II	ACE ID	ACE DD		
PCR-GoldMag LFA system	ACE II	127	0	0	127	100%
	ACE ID	0	132	0	132	100%
	ACE DD	0	0	41	41	100%

We established a sensitive, low cost, and easy-to-use large fragment insertion/deletion polymorphism detection platform using whole blood Direct PCR-LFA. This enabled us to accurately detect insertion/deletion polymorphisms for specific genetic diseases. With the help of gold magnetic nanoparticles and LFA, genotyping results could be rapidly visualized based on colored T and C lines.

Compared to conventional detection methods for I/D polymorphism, our assay had numerous significant advantages. These include (a) Using the PCR-GoldMag LFA system, it takes only 5 min to obtain results without the need for expensive or high-end instruments. (b) After sample treatment with NaOH, the whole testing procedure was rapid and convenient. (c) Previous methods were complex and performed on expensive and sophisticated instruments that may not be available in many laboratories, however, the PCR-GoldMag LFA method is easy-to-operate and affordable for on-site genotyping with high efficiency. Hence, this method could be run in laboratories that are not fully equipped with sophisticated instruments.

### Case-Control Study

Previous studies have reported that insertion/deletion (I/D) polymorphisms in the ACE gene were associated with cardiovascular and cerebrovascular diseases. However, the association between ACE gene I/D and cardiovascular and cerebrovascular diseases is controversial. Some studies have shown that ACE I/D polymorphisms are associated with coronary artery disease (CAD) and cerebral ischemic stroke (CIS) [14, 16], however, other studies have found no association [15, 17]. We performed a case-control study and a meta-analysis to evaluate the association between ACE I/D polymorphisms and coronary atherosclerotic heart disease, as well as stroke.

**Fig. 5:**
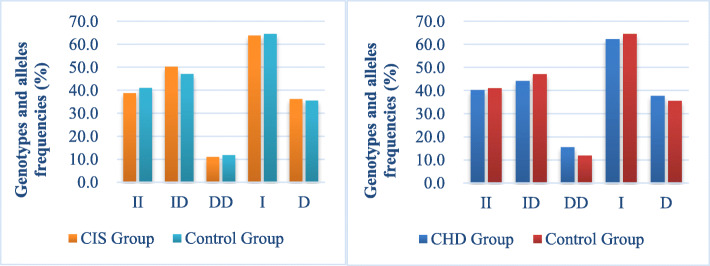
ACE genotype frequencies

We analyzed a total of 633 subjects (199 CHD patients, 207 CIS patients, and 227 control group) for the case-control study. These samples satisfied the Hardy–Weinberg Law (CHD, c2 = 0.68, *p* = 0.41 > 0.05; CIS, c2 = 1.58, *p* = 0.21 > 0.05), which signified a reliable representative group. Using the chi-square test, no statistical differences between these two groups were observed (Table 3-4). The ACE genotype frequencies are shown in Fig. 5. Based on statistical analysis, we found no association between ACE I/D polymorphisms and coronary heart disease or stroke. However, after grouping based on age, we observed a significant difference between the genotype and age of the patients in the CHD group (*p* = 0.02 < 0.05), with no significant differences in the stroke group (*p* = 0.07 > 0.05) (Table S2).

**Table 3:** Genotype and allele frequencies of ACE I/D polymorphism in coronary atherosclerotic heart disease and control group

	Marker		CHD Group (*n* = 199)	Control (*n* = 227)	χ2	*P*-Value
ACE I/D	Genotype	II	80	93	1.27	0.53
		ID	88	107		
		DD	31	27		
	Allele	I	248	293	0.36	0.55
		D	150	161

**Table 4:** Genotype and allele frequencies of ACE I/D polymorphism in the stroke and control group

	Marker		CIS Group (*n* = 207)	Control (*n* = 227)	χ2	*P*-Value
ACE I/D	Genotype	II	81	93	0.37	0.84
		ID	104	107		
		DD	23	27		
	Allele	I	266	293	0.01	0.91
		D	150	161

Association studies are influenced by selection bias, population stratification, confounding factors, and clinical criteria used to define patient groups. Future genetic association studies should include larger patient cohorts and strict study designs to determine potential associations between genetic susceptibility and cardiovascular and cerebrovascular diseases.

## Conclusions

Our genotyping method for determining ACE I/D polymorphism was established using a combination of Direct PCR and GoldMag-LFA system. This method used whole blood samples to directly perform PCR amplification. It eliminated the need for nucleic acid extraction and reduced the risk of sample cross-contamination during nucleic acid extraction. The method was rapid and sensitive and did not require expensive instruments. The whole PCR reaction took about 80 min and had significant clinical application. The genotyping results were obtained within 5 min after loading the PCR products onto the LFA device. The method could be used to determine I/D polymorphisms for a variety of genes that are associated with disease risk.

## Materials and Methods

### Materials and Reagents

GoldMag nanoparticles were purchased from Xi’an GoldMag Nanobiotech Co., Ltd. (Xi’an, China). 10 × HotMaster Taq Buffer and HotMaster Taq DNA Polymerase were purchased from TIANGEN (Beijing, China). dNTPs and uracil-DNA glycosylase (UDG) were purchased from Shinegene (Shanghai, China). Water (18.2 MΩ cm) was purified using the Barnstead Nanopure Water system. MgCl_2_ (25 mM) was purchased from Thermo Scientific (Shanghai, PRC). 1 × TE (pH = 8) were purchased from Sangon Biotech. All primers were synthesized and purchased from Invitrogen (Shanghai, China).

### LFA Device

Streptavidin and goat anti-mouse IgG were pre-immobilized in a test line (T line) and a control line (C line) onto a porous nitrocellulose membrane using the BioJet HM3010 dispenser (BioDot Inc., California, U.S.A.). Then, the probe solution containing the poly-acrylic acid (PAA) modified gold magnetic nanoparticles (PGMNPs) conjugated with anti-digoxin antibody was dispensed onto the conjugate pad of the LFA strips. These strips were dried and stored in a sealed aluminum foil bag at room temperature until required. The strips were stable for 12 months.

### Primer Design and Synthesis

Based on the ARMS-PCR method [33–35], the forward primer was designed as the universal primer, and the reverse primers were the allele-specific primers. Primer specificity was enhanced by introducing mismatches at the penultimate or antepenultimate at the 3′ end of the primers [36, 37]. For convenience, we refer to the wild-type as “WT” and the mutation type as “M”. To genotype the ACE gene, a set of three specific primers were designed using the Primer 5.0 software program (Primer-E Ltd., Plymouth, U.K.). This included the 5′ biotin-labeled universal primer, a 5′ digoxin labeled specific primer for WT, and a 5′ digoxin labeled specific primer for M. Two additional primers were designed for DNA sequencing. The primer sequences are shown in Table 5. All primers were synthesized by Invitrogen Biotechnology Ltd. (Shanghai, China).

**Table 5:** Primer sequences used for sequencing and PCR reactions

Allele	Application	Primer	Sequence (5′-3′)	Marker
ACE*16	Sequencing	*16-seq-F	CTGGAGAGCCACTCCCATCCTTTCT	
		*16-seq-R	GACGTGGCCATCACATTCGTCAGAT	
	PCR reactions	*16-F	AAGGAGAGGAGAGAGACTCAAGCAC	5′-labeled biotin
		*16-R (M)	TCGAGACCATCCCGGCTAAAAC	5′-labeled digoxin
		*16-R (WT)	AACCACATAAAAGTGACTGTATCGG	5′-labeled digoxin

### Sample Preparation

Whole blood is composed of hemoglobin and salt ions. DNA in whole blood is mainly derived from white blood cells. We treated fresh whole blood (in ethylenediaminetetraacetic acid (EDTA)-containing collection tube) with NaOH solution prior to performing whole blood direct PCR amplification. Fresh whole blood samples were harvested and then incubated at room temperature for 2 min with 100 mM NaOH at a 1:2 ratio. The volume of the mixture was 30 μL. Afterward, 5 μL of the mixture was used for subsequent amplification. The DNA template was not reusable and required immediate use. Whole blood samples stored at 4 °C for 1 week or frozen whole blood samples at − 20 °C could be used for the assay. Samples should not be freeze-thawed for more than 3 cycles.

### PCR Amplification

The PCR amplification was performed in two separate PCR tubes (M tube and WT tube). Both PCR reactions were run simultaneously using the same template. The final PCR reaction volume was 50 μL, and included 5 μL of 10 × HotMaster Taq Buffer (10 mM Tris HCl, 50 mM KCl), 200 μM of each dNTPs (dATP, dCTP, dGTP, dUTP), 3 μL of MgCl_2_ (25 mM), 0.5 U of Hotmaster Taq DNA polymerase, 0.5 U of UDG polymerase, a specific concentration of primers which included the common and specific primer (R (M) primer in M tube and R (WT) primer in WT tube), and 5 μL of prepared fresh whole blood. PCR amplifications were performed using a 2720 Thermal Cycler (Applied Biosystems, Foster City, U.S.A.). In this systerm, we have introduced an dUTP-UNG strategy to prevent carryover contamination. So whole blood direct PCR amplification was performed with two initial denaturation steps, 2 min at 50 °C for elimination of residual PCR product contamination using UNG enzyme [38] and 3 min 30s at 95 °C for inactivation of UNG [39], followed by 31 cycles of denaturation at 94 °C for 5 s, annealing at 60 °C for 10 s, and extension at 65 °C for 30 s. The final extension step was at 65 °C for 10 min, and then a hold at 4 °C.

### Clinical Application and Statistical Analysis

We analyzed 300 clinical whole blood samples obtained from the People’s Hospital of Shaanxi Province. Appropriate ethical and governance permission was obtained from the local authorities prior to blood sample collection. ACE genotyping was performed using the Direct PCR-LFA system and then compared using 2% agarose gel electrophoresis. In addition, 100 case samples were randomly selected for DNA sequencing. DNA for agarose gel electrophoresis was extracted from blood samples using the whole blood genomic DNA isolation kit purchased from Xi’an GoldMag Nanobiotech Co., Ltd. (Xi’an, Shaanxi, PRC). In addition, we analyzed additional samples to determine the accuracy of our method. Statistical analysis was performed based on the results of the two methods.

## Supplementary Information

**Additional file 1: Figure S1.** Optimization of the detection system. M = M tube. WT = WT tube. 1=II 2=ID 3=DD (**A**) The cycles of PCR amplification. 31 cycles were the optimal. (**B**) The annealing temperature. 60°C was found to be optimal. (**C**) The concentration of primers. 2.5 μM primer was proved to be optimal. (**D**) The test of the amount of Mg^2+^ with 3 μL of Mg^2+^ as the optimum. (**E**) The amount of the whole blood template. 5 μL was optimal. **Figure S2.** Partial genotyping results of the two methods. (**A**) The results of 20 cases of whole blood direct PCR (**B**) Agarose gel electrophoresis. **Figure S3.** Test results of special samples. 1-5: High bilirubin sample 6-10: Autoimmune Disease sample 11-15: Low white blood cell concentration sample 16-20: High white blood cell concentration sample 21-25: High cholesterol sample 26-30: High triglyceride sample 31-35: Hemolysis sample 36-40: Blood disease sample. **Table S1.** Comparison of nucleic acid quantities and Comparison of nucleic acid quantities. **Table S2.** Association of ACE (I/D) polymorphism with age of study subjects.

## Data Availability

All data generated or analyzed during this study are included in this published article.
