# Supplementary material for: A Novel PCR Method for Detecting ACE Gene Insertion/Deletion Polymorphisms and its Clinical Application
Source: Biol Proced Online. 2021 Jan 7;23:2. doi: 10.1186/s12575-020-00140-6 (PMC7791644; doi:10.1186/s12575-020-00140-6)
Supplement: Supplementary file 1 — Additional file 1: Figure S1. Optimization of the detection system. M = M tube. WT = WT tube. 1=II 2=ID 3=DD (A) The cycles of PCR amplification. 31 cycles were the optimal. (B) The annealing temperature. 60°C was found to be optimal. (C) The concentration of primers. 2.5 μM primer was proved to be optimal. (D) The test of the amount of Mg2+ with 3 μL of Mg2+ as the optimum. (E) The amount of the whole blood template. 5 μL was optimal. Figure S2. Partial genotyping results of the two methods. (A) The results of 20 cases of whole blood direct PCR (B) Agarose gel electrophoresis. Figure S3. Test results of special samples. 1-5: High bilirubin sample 6-10: Autoimmune Disease sample 11-15: Low white blood cell concentration sample 16-20: High white blood cell concentration sample 21-25: High cholesterol sample 26-30: High triglyceride sample 31-35: Hemolysis sample 36-40: Blood disease sample. Table S1. Comparison of nucleic acid quantities and Comparison of nucleic acid quantities. Table S2. Association of ACE (I/D) polymorphism with age of study subjects. [file 12575_2020_140_MOESM1_ESM.docx]

Supplement Materials

**ABSTRACT**

Angiotensin-converting enzyme (ACE) plays a major role in blood pressure regulation and cardiovascular homeostasis. The wide distribution and multifunctional properties of ACE suggest it’s involvement in various pathophysiological conditions. In this study, a novel visual detection method for ACE I/D polymorphisms was designed by integrating direct PCR without the need for DNA extraction using gold magnetic nanoparticles (GMNPs)-based lateral flow assay (LFA) biosensor. The entire detection procedure could enable the genotyping of clinical samples in about 80 minutes. The detection limit was 0.75 ng and results could be obtained in 5 minutes using the LFA device. Three hundred peripheral blood samples were analyzed using the direct PCR-LFA system and then verified by sequencing to determine accuracy and repeatability. A clinical preliminary study was then performed to analyze a total of 633 clinical samples. After grouping based on age, we found a significant difference between the genotypes and the age of patients in the CHD group. The introduction of this method into clinical practice may be helpful for the diagnosis of diseases caused by large fragment gene insertions/deletions.


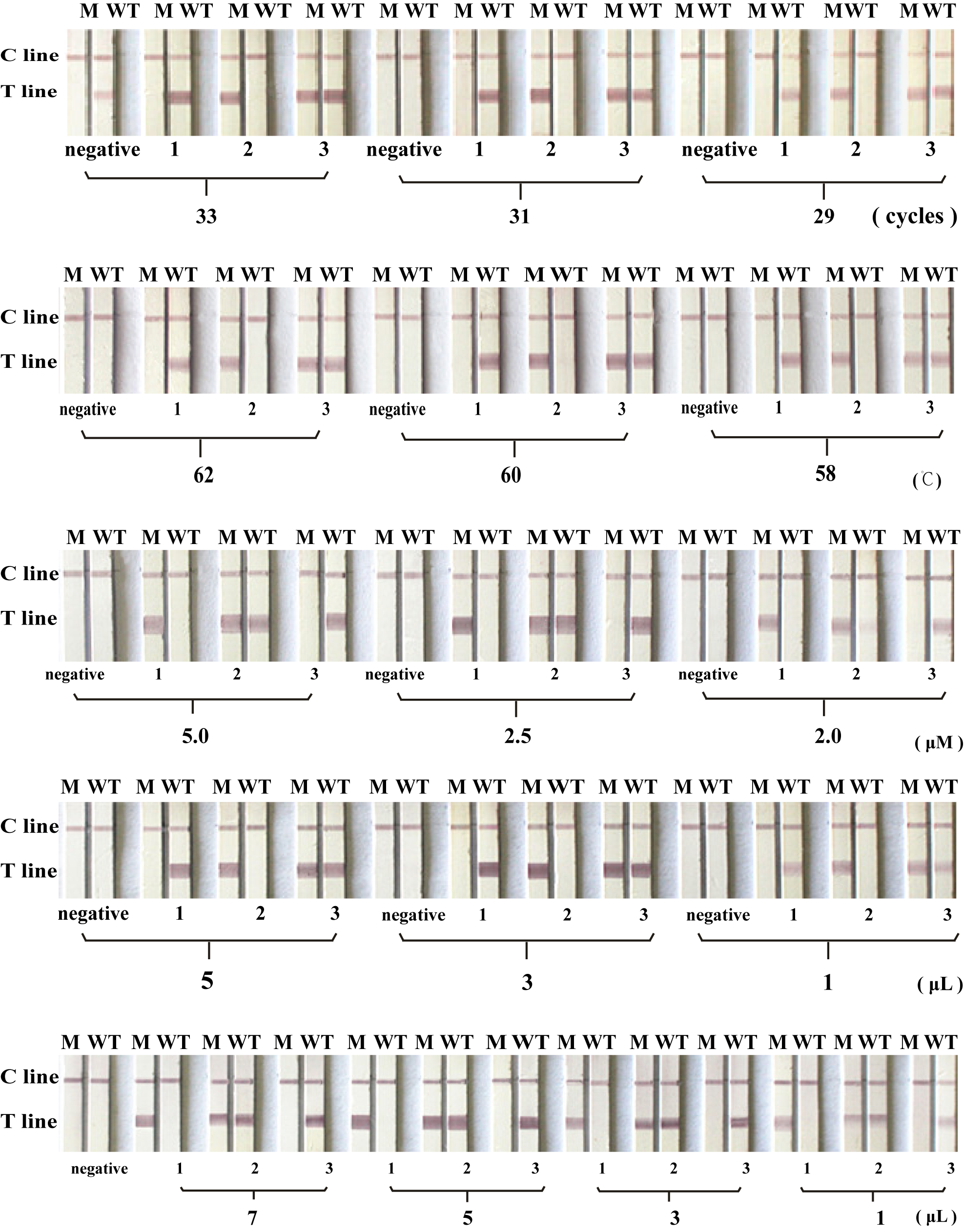


**Figure. S1** Optimization of the detection system. M = M tube. WT = WT tube. 1=II 2=ID 3=DD (**A**) The cycles of PCR amplification. 31 cycles were the optimal. (**B**) The annealing temperature. 60°C was found to be optimal. (**C**) The concentration of primers. 2.5 μM primer was proved to be optimal. (**D**) The test of the amount of Mg^2+^ with 3 μL of Mg^2+^ as the optimum. (**E**) The amount of the whole blood template. 5 μL was optimal.


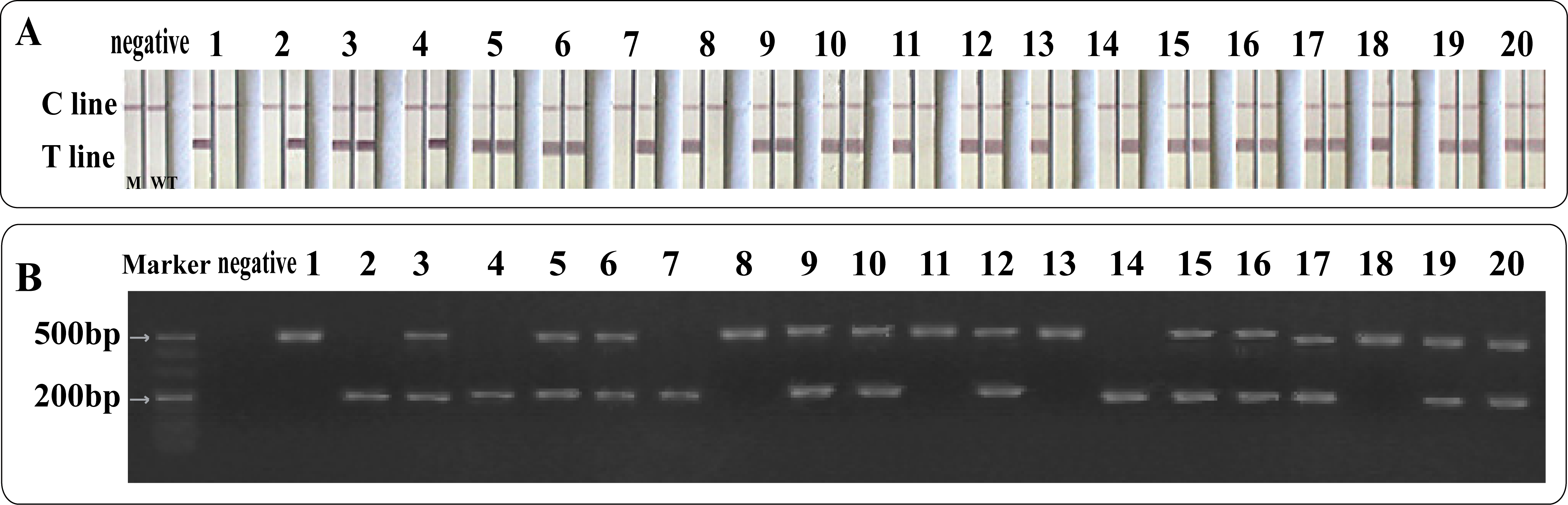


**Figure. S2** Partial genotyping results of the two methods. (**A**) The results of 20 cases of whole blood direct PCR (**B**) Agarose gel electrophoresis


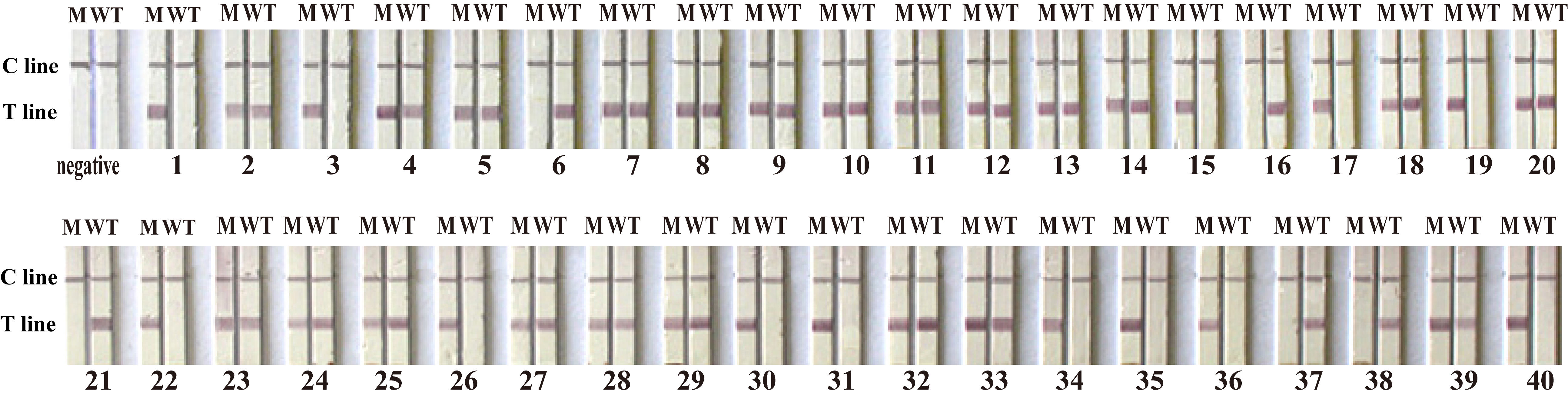


**Figure. S3** Test results of special samples. 1-5: High bilirubin sample 6-10: Autoimmune Disease sample 11-15: Low white blood cell concentration sample 16-20: High white blood cell concentration sample 21-25: High cholesterol sample 26-30: High triglyceride sample 31-35: Hemolysis sample 36-40: Blood disease sample

**Table. S1** Comparison of nucleic acid quantities and Comparison of nucleic acid quantities

| Comparison item | Sample type | Dilution ratio | | | | | | | | | | |
| --- | --- | --- | --- | --- | --- | --- | --- | --- | --- | --- | --- | --- |
|  |  | 1:1 | 1:2 | 1:4 | 1:5 | 1:10 | 1:15 | 1:20 | 1:30 | 1:40 | 1:60 | 1:120 |
| White blood cell count | Homozygous deletion sample | 5.34 | 2.7 | 1.4 | 1.2 | 0.5 | 0.4 | 0.27 | 0.18 | 0.13 | 0.08 | 0.03 |
|  | Insert/deletion heterozygous sample | 11.9 | 5.9 | 3.1 | 4.3 | 1.17 | 0.94 | 0.6 | 0.49 | 0.28 | 0.2 | 0.13 |
|  | Homozygous insertion sample | 4.64 | 2.3 | 1.2 | 0.99 | 0.47 | 0.31 | 0.23 | 0.15 | 0.11 | 0.07 | 0.04 |
| The amount of nucleic acid extracted from blood samples | Homozygous deletion sample | 367 | 289 | 251.5 | 233.5 | 213 | 199.5 | 123.5 | 108 | 66.5 | 57.5 | 28.5 |
|  | Insert/deletion heterozygous sample | 209.25­­ | 114.9 | 100.8 | 60.6 | 36.9 | 36.15 | 33.9 | 30.9 | 29.4 | 26.1 | 13.2 |
|  | Homozygous insertion sample | 207.5 | 184.5 | 166 | 180.5 | 162.5 | 149.5 | 126.5 | 98 | 74 | 52,5 | 32.5 |
| The amount of nucleic acid converted by direct blood expansion | Homozygous deletion sample | 49.84 | 25.2 | 13.07 | 11.2 | 4.67 | 3.73 | 2.52 | 1.68 | 1.21 | 0.75 | 0.28 |
|  | Insert/deletion heterozygous sample | 111.72 | 55.07 | 28.93 | 40.13 | 10.92 | 8.77 | 5.6 | 4.57 | 2.61 | 1.87 | 1.21 |
|  | Homozygous insertion sample | 43.31 | 21.47 | 11.2 | 9.24 | 4.39 | 2.89 | 2.15 | 1.40 | 1.03 | 0.65 | 0.37 |

**Table. S2** Association of ACE(I/D) polymorphism with age of study subjects

|  |  | ACE (I/D) Polymorphism | | | P Value |
| --- | --- | --- | --- | --- | --- |
| Groups | Age Grouping | DD | ID | II |  |
| CHD | 31-50 | 2 | 8 | 17 | 0.02 |
|  | 51-70 | 22 | 72 | 49 |  |
|  | 71-90 | 7 | 8 | 14 |  |
| CIS | 31-50 | 1 | 15 | 11 | 0.07 |
|  | 51-70 | 14 | 76 | 62 |  |
|  | 71-90 | 8 | 13 | 8 |  |
| Control | 31-50 | 2 | 11 | 8 | 0.57 |
|  | 51-70 | 21 | 88 | 72 |  |
|  | 71-90 | 4 | 8 | 13 |  |

­
